# Assessment of humoral immune response to two mRNA SARS‐CoV‐2 vaccines (Moderna and Pfizer) in healthcare workers fully vaccinated with and without a history of previous infection

**DOI:** 10.1111/jam.15699

**Published:** 2022-07-18

**Authors:** Laura Serrano, Sonia Algarate, Beatriz Herrero‐Cortina, Jessica Bueno, María T. González‐Barriga, María Ducons, Jesica Montero‐Marco, Beatriz Acha, Ana Taboada, Pilar Sanz‐Burillo, Cristina Yuste, Rafael Benito, RIPOVAC Study Group

**Affiliations:** ^1^ Occupational Health Unit Lozano Blesa University Hospital Zaragoza Spain; ^2^ Institute for Health Research Aragón Zaragoza Spain; ^3^ Microbiology Department Lozano Blesa University Hospital Zaragoza Spain; ^4^ Microbiology Department Zaragoza University Zaragoza Spain; ^5^ Investigation Unit Lozano Blesa University Hospital Zaragoza Spain; ^6^ Universidad San Jorge Villanueva de Gállego Zaragoza Spain

**Keywords:** anti‐S IgG SARS‐CoV‐2, healthcare workers, immune response, Moderna, mRNA SARS‐CoV‐2 vaccines, Pfizer, post‐vaccination, RIPOVAC

## Abstract

**Aims:**

Presence of anti‐S1 region of SARS‐CoV‐2 spike protein was analysed, at two and eight months, in 477 immunocompetent healthcare workers in Zaragoza, Spain, vaccinated with mRNA‐1273 (Moderna) or BNT162b2 (Pfizer).

**Methods and results:**

Antibody analysis was performed with Alinity i System (Abbott). At 2 months, 100% of vaccinated had anti‐S1 IgG (mean = 13,285 AU ml^−1^). This value was significantly higher with Moderna (18,192 AU ml^−1^) than with Pfizer (10,441 AU ml^−1^). The mean value of anti‐S1 IgG after vaccination was significantly higher in patients with than without previous infection (18,539 vs. 7919 AU ml^−1^); in both groups was significantly higher with Moderna than with Pfizer (21,881 vs. 15,733 AU ml^−1^ and 11,949 vs. 6387 AU ml^−1^), respectively. At 8 months, 100% of patients were IgG positive, with higher levels with Moderna than with Pfizer. Nevertheless, in ensemble of cases, a mean decrease of antibody levels of 11,025 AU ml^−1^ was observed.

**Conclusion:**

At 2 and 8 months after vaccination, IgG response persists with both vaccines but with important decrease which suggests the need for revaccination.

**Significance and impact of study:**

The study contributes to know the immune status after vaccination with two of more used anti‐SARS‐CoV‐2 vaccines. This knowledge is important for establishing the best vaccination strategy

## INTRODUCTION

The recent rollout of vaccines that induce specific antibodies to SARS‐CoV‐2 has provided an important tool in controlling the pandemic.

The serology of SARS‐CoV‐2, in addition to being a complementary tool in the diagnosis (Fuentes et al., 2021), allows knowing the immune status of the patient after natural infection or after vaccination (Okba et al., 2020).

SARS‐CoV‐2 induces antibody formation against S (spike) and N (nucleocapsid) proteins. Anti‐S1 IgG may have neutralizing activity. This activity can be evaluated in vitro with the plate reduction neutralization test, which is difficult to be applied to routine (Santiago et al., 2021). For this reason, diagnostic tests have been manufactured for the quantitative measurement of these antibodies to establish the degree and duration of immunity and, based on them, to develop preventive vaccination strategies.

We present data from the observational and prospective RIPOVAC study in a group of healthcare workers in Aragón (Spain). This study aims to quantify the response of IgG against the region of the S1 subunit of the SARS‐CoV‐2 spike protein that binds to the receptor‐binding domain (RBD) (anti‐S1 IgG) and of IgM against the spike protein (anti‐S IgM), two and 8 months after vaccination with two doses of two first‐generation mRNA vaccines, Moderna (mRNA‐1273) or Pfizer‐BioNTech (BNT162b2) (with the interval time recommended by the manufacturer, 28 or 21 days, respectively), and the influence on it of pre‐vaccination SARS‐CoV‐2 infection.

## MATERIALS AND METHODS

We show data from the RIPOVAC study, conducted at the Lozano Blesa University Clinical Hospital of Zaragoza, Spain, the reference centre of Sector III of the Aragón Health Service (SALUD). Initially, 494 cases were included, belonging to all categories of healthcare workers, but 17 (3.44%) were lost. Finally, 477 voluntary, immunocompetent, non‐pregnant healthcare workers (71 male and 406 female, mean age of 45,29 years) vaccinated with Moderna or Pfizer, according to the availability, between February and March 2021 were included. Cases with SARS‐CoV‐2 infection between the first dose and the immunogenicity analysis were excluded.

The first post‐vaccine sample was obtained from 477 healthcare workers [Moderna 175 (36.69%) and Pfizer 302 (63.31%)] 2 months after the second dose ±7 days (months of April, May and June 2021), shortly after the peak of antibodies was reached (Montoya et al., 2021).

Table 1 shows the distribution of 477 healthcare workers by sex, age, type of vaccine and existence or not of previous infection. Women predominate in both groups vaccinated with Moderna and Pfizer, but without significant differences in age.

**TABLE 1:** Characteristics of 477 enrolled healthcare workers

Vaccine type	Sex (%) mean age (SD)	Previous infection Yes No	
		Yes	No
Moderna (175)			
	28 male (16.00%) 42.14 (13.59)	22 (12.57%)	6 (3.43%)
	147 female (84.00%) 43.31 (11.94)	88 (50.29%)	59 (33.71%)
Pfizer (302)			
	43 male (14.24%) Age: 45.71 (SD 12.36)	23 (7.62%)	20 (6.62%)
	259 female (85.76%) Age: 46.77 (SD 11.57)	108 (35.76%)	151 (50.00%)

Second sample was obtained from 444 workers, 53 male y 391 female, with mean age of 45,61 years [Moderna 166 (37.39%) and Pfizer 278 (62.61%)] 8 months after the second dose.

People with previous positive PCR to SARS‐CoV‐2 (Alinity m, Abbott; Viasure, Certest Biotec; or GeneXpert, Cepheid), or with previous positive SARS‐CoV‐2 antigen (PanBio COVID‐19 Ag, Abbott) or with presence of anti‐N IgG (SARS‐CoV‐2 IgG, Abbott) were considered previously infected.

The determination of quantitative anti‐S1 IgG (AU ml^−1^) and anti‐S IgM (index) was performed by CMIA (SARS‐CoV‐2 IgG II Quant, Abbott and SARS‐CoV‐2 IgM, Abbott, respectively) by blinded, trained laboratory staff, using the Abbott Alinity i platform, following the manufacturer's instructions. These reagent kits have a specificity of 100% and sensitivity at 15 days of 98.77%, according to the manufacturer.

The analysis was carried out based on the vaccine received, Moderna (175 cases) or Pfizer (302 cases), and the existence or not of a previous SARS‐CoV‐2 infection.

The protocol was approved by the Clinical Investigation Ethics Committee of Aragón (EPA 21/000) and the recruited healthcare workers gave their written informed consent, following the guidelines of the Declaration of Helsinki.

### Statistical analysis

Quantitative variables were described using the mean (standard deviation) and categorical variables were reported using frequencies. Differences in sociodemographic and serological data between the groups were tested using the independent student's *t*‐test or the Mann–Whitney *U*‐test and reported as mean difference (95% confidence interval, CI). Statistical significance was set at *p* < 0.05 for all calculations. Data analysis was performed using SPSS v.19 (IBM, Chicago, IL, USA).

## RESULTS

All 477 recruited healthcare workers were anti‐S1 IgG positive at 2 months after vaccination, regardless of the type of vaccine received. The responses ranged from 200 to >40,000 AU ml^−1^, with a mean of 13,285 AU ml^−1^ (95% CI: 12,317 to 14,253) (Table 2). Only 5 cases (1.05%) had anti‐S1 IgG values <1000 AU ml^−1^.

**TABLE 2:** IgG and IgM response in total population and vaccine type

Vaccine	Patients	IgG ANTI‐S1			IgM ANTI‐S		
		Positive	Mean AU ml^−1^ (SD)	Range	Positive	Mean index (SD)	Range
Moderna	175	175 (100%)	18,192 (11556)	2111–>40,000	41 (23.43%)	3.06 (2.76)	1.03–12.28
Pfizer	302	302 (100%)	10,441 (9144)	200–>40,000	69 (22.85%)	2.89 (2.27)	1.00–14.93
Total	477	477 (100%)	13,285 (10755)	200–>40,000	110 (23.06%)	2.95 (2.45)	1.00–14.93

Nevertheless, anti‐S IgM was only positive in 110 cases (23.06%), with a mean index of 2.95 (SD 2.45, range 1.00 to 14.93) (Table 2). 50% of the anti‐S IgM positive cases had indexes between 1.00 and 2.00.

In Moderna‐vaccinated group (175 cases), the anti‐S1 IgG mean was 18,192 AU ml^−1^ (95% CI 16,468 to 19,917), significantly higher than in the group vaccinated with Pfizer (10,441 AU ml^−1^, 95% CI 9406 to 11,477). Mean difference between Moderna and Pfizer was 7751 (95% CI: 5866 to 9635) (*p* < 0.001). The same performance was seen for IgM, although without significant differences, showing a higher percentage of positivity and a higher mean index for Moderna than for Pfizer, with percentages of 23.43% and 22.85%, and average indexes of 3.06 (95% CI: 2.18 to 3.83) and 2.89 (95% CI: 2.35 to 3.33), respectively (Table 2).

In cases without previous infection, anti‐S1 IgG response was significantly more intense with Moderna (11,949 AU ml^−1^, 95% CI, 9974 to 13,924) than with Pfizer (6387 AU ml^−1^, 95% CI: 5579 to 7196). Mean difference between Moderna and Pfizer was 5562 (95% CI: 3787 to 7336) (*p* < 0.001). Anti‐S1 IgM response was also greater both in terms of percentage and mean values (Table 3), but without significant differences.

**TABLE 3:** IgG and IgM response in total population and vaccine type in non‐infected patients

Vaccine	Patients	IgG anti‐S1			IgM anti‐S		
		Positive	Mean AU ml^−1^ (SD)	Range	Positive	Mean index (SD)	Range
Moderna	65	65 (100%)	11,949 (7971)	2111–>40,000	8 (12.30%)	4.83 (4.23)	1.32–12.28
Pfizer	171	171 (100%)	6387 (5355)	715–36,122	19 (11.11%)	2.63 (1.95)	1.20–7.73
Total	236	236 (100%)	7919 (6651)	715–>40,000	27 (11.44%)	3.28 (2.91)	1.20–12.28

In cases with history of previous infection, anti‐S1 IgG response with Moderna vaccine has been significantly more intense, with a mean value of 21,881 AU ml^−1^ (95% CI: 19,655 to 24,109) versus 15,733 AU ml^−1^ with Pfizer (95% CI: 13,951 to 17,515). Mean difference between Moderna y Pfizer was 6149 (95% CI: 3344 to 8953) (*p* < 0.001) (Table 4). The response in cases with previous infection is higher than in patients without previous infection.

**TABLE 4:** IgG and IgM response in total population and vaccine type in ex‐infected patients

Vaccine	Patients	IgG anti‐S1			IgM anti‐S		
		Positive	Mean AU ml^−1^ (SD)	Range	Positive	Mean index (SD)	Range
Moderna	110	110 (100%)	21,881 (11786)	3335–>40,000	33 (30.00%)	2.62 (2,14)	1.03–12.13
Pfizer	131	131 (100%)	15,733 (10310)	200–>40,000	50 (38.16%)	2.98 (2.39)	1.00–14.93
Total	241	241 (100%)	18,539 (11405)	200–>40,000	83 (34.43%)	2.84 (2.29)	1.00–14.93

In contrast, the anti‐S IgM response has been stronger after the Pfizer vaccine, both in percentage and mean terms, but without significant differences (Table 4).

The 444 healthcare workers analysed 8 months after the second dose also present anti‐S, with a mean value of 4297 AU ml^−1^ with Moderna (SD 6289; range 132–>40,000) and of 3449 with Pfizer (SD 6645; range 120–>40,000) (Table 5). Antibody levels with Moderna were significantly higher than with Pflzer, also 8 months after vaccination. (*p* < 0.001).

**TABLE 5:** IgG anti‐S1 response 8 months after the second dose of vaccine

Vaccine	Patients	Positive	Mean AU ml^−1^ (SD)	Range
Moderna	166	166 (100%)	4297 (6289)	132–>40,000
Pfizer	278	278 (100%)	3449 (6645)	120–>40,000
Total	444	444 (100%)	3766 (6519)	120–>40,000

Of the 444 health workers who were analysed at eight months, antibody levels had diminished in 416 (93.69%) and increased in 25 (5.63%). At least four of the latter had been infected after the first extraction (PCR or positive anti‐N IgG). In the remaining 3 (0.68%), no change could be demonstrated, because both samples presented an antibody level > 40,000 AU ml^−1^ (Table 6).

**TABLE 6:** Antibody level variation 8 months after the second dose of vaccine

Variation	Number of cases	%
Decrease	416	93.69
Increase	25	5,53
No change	3	0,68

At 8 months, antibody level decreased by a mean of 11,025 AU ml^−1^, regardless of the type of vaccine administered and 120 cases (72.29%) out of vaccinated with Moderna and 159 cases (57.19%) out of vaccinated with Pfizer showed antibody levels >=1000 AU ml^−1^. Antibody levels at 8 months after vaccination had decreased to 23.62% and 33.03% in Moderna or Pfizer vaccinated, respectively.

In people younger than 30 years, antibody levels are higher compared to older patients (4229 vs. 3699) at 8 months and decrease faster, regardless of the type of vaccine received.

## DISCUSSION

The reagents used for the determination of anti‐S and anti‐N antibodies have been evaluated by various authors (Bryan et al., 2020; Narasimhan et al., 2021) with satisfactory results. In the case of IgG, the kit provides quantitative results expressed in AU ml^−1^.

Pfizer vaccine confers 95% protection against COVID‐19 (Polack et al., 2020). Similar data have been showed by other authors (Vasileiou et al., 2021). Moderna vaccine has a 94.1% efficacy in preventing SARS‐CoV‐2 disease, including severe disease (Baden et al., 2021; Mahase, 2020).

Both vaccines induce the production of antibodies to the spike (S protein) that correlate significantly with neutralizing activity for SARS‐CoV‐2 (Wajnberg et al., 2020). Both have been applied to healthcare workers in Sector III of the SALUD in Aragon, Spain.

Knowledge of the kinetics of antibodies induced by vaccines against COVID is important to implement a more appropriate preventive strategy, especially when vaccines may be less effective against variants of the virus (Jangra et al., 2021).

The Moderna and Pfizer vaccines induced an IgG response in 100% of the studied cases, which is still present 2 months after the second dose, although with different levels of intensity. Moderna vaccine induces a higher response in terms of mean IgG concentration, percentage of positive IgM and average IgM index. This applies to patients without and with previous infection.

These data would be consistent with those published by several authors (Krammer et al., 2021; Manisty et al., 2021), who observed a significantly higher IgG response following Pfizer vaccine in patients with previous infection. In addition, Montoya et al. (2021) describe significant differences in IgG indexes in favour of Moderna between 42–48 and 70–83 days after the second dose.

In five cases, all of them women, from 37 to 55 years old, anti‐S1 IgG values were less than 1000 AU ml^−1^, lower than the average value of all the healthcare workers studied. Four of them had not been previously infected and had numbers greater than 700. The fifth had an IgG concentration of 200.7 AU ml^−1^: this case had been previously asymptomatically infected and with anti‐N IgG indexes of 5.13 and 4.97 at 11 and 7 months prior to sampling, respectively, and 5.07 at the time of serum extraction for this study.

The low percentage of IgM positive cases 2 months after vaccination with both types of vaccines is surprising. It is probably a reproduction of low IgM response after vaccination with rapid negativization or even without IgM response. This is a similar situation to that described in 20% of MERS patients in whom antibodies are not detected at 30 days of evolution (Corman et al., 2016). In other study, all patients formed antibodies, but 28% were IgM negative at 2 months (Shi et al., 2004). In the study by Narasimhan et al. (2021) IgM was detected until day 80, but with oscillating values from day 23 with a downward trend.

The IgM response has been stronger with Pfizer, in percentage and mean, in patients who had been infected, but without significant differences. There may be a bias in the classification of cases without prior infection owing to a short persistence of anti‐N IgG in cases of previous infection with SARS‐CoV‐2 (Shang et al., 2021).

These differences in the response of the immune system may be related with the different concentration of vaccine antigen and administration regimen, with Moderna 100 micrograms and boosted 4 weeks after and with Pfizer 30 micrograms and boosted 3 weeks later.

The stronger response with Moderna vaccine, in patients without previous infection and in previously infected patients, may point to a greater persistence of antibodies induced by it and represents greater immunoprotection.

IgG antibody levels anti‐SARS‐CoV2 persist at 8 months in all healthcare workers after complete vaccination with Moderna or Pfizer, but decreasing to 23% and 33%, respectively. The faster decrease of antibody levels with Moderna could be related with a more intense initial response. These data suggest a limited persistence of antibodies after vaccination and the need for revaccination. In patients with a mild or moderate disease course, immunological response appears in most individuals and persists for at least 10 months, although both IgG antibody levels and IFN‐γ concentrations decreased to about a half within 300 days (Schiffner et al., 2021).

In people under 30 years, the antibody levels were higher after 8 months and diminished faster. It could be explained because the younger produced more antibodies after the first determination compared to the older because the immune system is less effective as age increases.

More studies are needed to determine the duration of response and the level of protection, important data to determine the frequency of revaccination, the antigenic formulation and the efficacy of vaccines against the different variants.

## AUTHOR CONTRIBUTIONS

L.S., and R.B. designed and coordinated the research studies, conducted experiments, analysed data, and wrote the first version of the manuscript; S.A., J.B., and M.D. conducted experiments; B.H., MT.G., J.M., B.A., A.T., P.S., and C.Y. vaccinated, got data and registered the subjets and obtained human samples. All authors have read and agreed to the published version of the manuscript.

## CONFLICT OF INTEREST

The authors declare no conflict of interest.

## Data Availability

Data available on request from the authors.

## References

[jam15699-bib-0001] Baden, L.R. , El Sahly, H.M. , Essink, B. , Kotloff, K. , Frey, S. , Novak, R. et al. (2021) Efficacy and safety of the mRNA‐1273 SARS‐Cov‐2 vaccine. New England Journal of Medicine, 384, 403–416. 33378609 10.1056/NEJMoa2035389 PMC7787219

[jam15699-bib-0002] Bryan, A. , Pepper, G. , Wener, M.H. , Fink, S.L. , Morishima, C. , Chaudhary, A. et al. (2020) Performance characteristics of the Abbott architect SARS‐CoV‐2 IgG assay and seroprevalence in Boise, Idaho. Journal of Clinical Microbiology, 58, e00941–e00920. 32381641 10.1128/JCM.00941-20 PMC7383515

[jam15699-bib-0003] Corman, V.M. , Albarrak, A.M. , Omrani, A.S. , Albarrak, M.A. , Farah, M.E. , Almasri, M. et al. (2016). Viral shedding and antibody response in 37 patients with Middle East respiratory syndrome coronavirus infection. Clinical Infectious Diseases, 62, 477–483. 26565003 10.1093/cid/civ951 PMC7108065

[jam15699-bib-0005] Fuentes, A. , Serrano‐Conde, E. , Roldán, C. , Benito‐Ruesca, R. , Mejías, G. , Sampedro, A. et al. (2021) Antibody response in patients admitted to the hospital with suspected SARS‐CoV‐2 infection: results from a multicenter study across Spain. European Journal of Clinical Microbiology and Infectious Diseases, 29, 1–7. 10.1007/s10096-020-04139-5 PMC7843881 33512616

[jam15699-bib-0006] Jangra, S. , Ye, C. , Rathnasinghe, R. , Stadlbauer, D. , Krammer, F. , Simon, V. et al. (2021) SARS‐CoV‐2 spike E484K mutation reduces antibody neutralisation. Lancet Microbe, 2, e283–e284. 33846703 10.1016/S2666-5247(21)00068-9 PMC8026167

[jam15699-bib-0007] Krammer, F ., Srivastava, K. , the PARIS team , Simon, V . (2021) Robust spike antibody 1 responses and increased reactogenicity in seropositive individuals after a single dose of SARS‐CoV‐2 mRNA vaccine. *medRxiv*. 10.1101/2021.01.29.21250653.

[jam15699-bib-0008] Mahase, E. (2020) Covid‐19: moderna applies for US and EU approval as vaccine trial reports 94.1% efficacy. British Medical Journal, 371, m4709. 33268462 10.1136/bmj.m4709

[jam15699-bib-0009] Manisty, C. , Otter, A.D. , Treibel, T.A. , McKnight, A. , Altmann, D.M. , Brooks, T. et al. (2021) Antibody response to first BNT162b2 dose in previously SARS‐CoV‐2‐infected individuals. Lancet, 397, 1057–1058. 33640038 10.1016/S0140-6736(21)00501-8 PMC7972310

[jam15699-bib-0010] Montoya, J.G. , Adams, A.E. , Bonetti, V. , Deng, S. , Link, N.A. , Pertsch, S. et al. (2021) Differences in IgG antibody responses following BNT162b2 and 2 mRNA‐1273 vaccines. Microbiology Spectrum, 9, e0116221. 34756093 10.1128/Spectrum.01162-21 PMC8579939

[jam15699-bib-0011] Narasimhan, M. , Mahimainathan, L. , Araj, E. , Clark, A.E. , Markantonis, J. , Green, A. et al. (2021) Clinical evaluation of the Abbott Alinity SARS‐CoV‐2 spike‐specific quantitative IgG and IgM assays among infected, recovered, and vaccinated groups. Journal of Clinical Microbiology, 59, e0038821. 33827901 10.1128/JCM.00388-21 PMC8218760

[jam15699-bib-0012] Okba, N.M.A. , Müller, M.A. , Li, W. , Wang, C. , GeurtsvanKessel, C.H. , Corman, V.M. et al. (2020) Severe acute respiratory syndrome coronavirus 2‐specific antibody responses in coronavirus disease patients. Emerging Infectious Diseases, 26, 1478–1488. 32267220 10.3201/eid2607.200841 PMC7323511

[jam15699-bib-0013] Polack, F.P. , Thomas, S.J. , Kitchin, N. , Absalon, J. , Gurtman, A. , Lockhart, S. et al. (2020) Safety and efficacy of the BNT162b2 mRNA Covid‐19 vaccine. New England Journal of Medicine, 383, 2603–2615. 33301246 10.1056/NEJMoa2034577 PMC7745181

[jam15699-bib-0014] Santiago, L. , Uranga‐Murillo, I. , Arias, M. , González‐Ramírez, A.M. , Macías‐León, J. , Moreo, E. et al. (2021) Determination of the concentration of IgG against the spike receptor‐binding domain that predicts the viral neutralizing activity of convalescent plasma and serum against SARS‐CoV‐2. Biology (Basel), 10, 208. 33801808 10.3390/biology10030208 PMC8001978

[jam15699-bib-0015] Schiffner, J. , Backhaus, I. , Rimmele, J. , Schulz, S. , Möhlenkamp, T. , Klemens, J.M. et al. (2021) Long‐term course of humoral and cellular immune responses in outpatients after SARS‐CoV‐2 infection. Frontiers in Public Health, 27, 732787. 10.3389/fpubh.2021.732787 PMC8502872 34646805

[jam15699-bib-0016] Shang, Y.F. , Liu, T. , Yu, J.N. , Xu, X.R. , Zahid, K.R. , Wei, C. et al. (2021) Half‐year follow‐up of patients recovering from severe COVID‐19: analysis of symptoms and their risk factors. Journal of Internal Medicine, 290, 444–450. 33904618 10.1111/joim.13284 PMC8242565

[jam15699-bib-0017] Shi, Y. , Wan, Z. , Li, L. , Li, P. , Li, C. , Ma, Q. et al. (2004) Antibody responses against SARS coronavirus and its nucleocapsid in SARS patients. Journal of Clinical Virology, 31, 66–68. 15288616 10.1016/j.jcv.2004.05.006 PMC7129167

[jam15699-bib-0018] Vasileiou, E. , Simpson, C.R. , Shi, T. , Kerr, S. , Agrawal, U. , Akbari, A. et al. (2021) Interim findings from first‐dose mass COVID‐19 vaccination roll‐out and COVID‐19 hospital admissions in Scotland: a national prospective cohort study. Lancet, 397, 1646–1657. 33901420 10.1016/S0140-6736(21)00677-2 PMC8064669

[jam15699-bib-0019] Wajnberg, A. , Amanat, F. , Firpo, A. , Altman, D.R. , Bailey, M.J. , Mansour, M. et al. (2020) Robust neutralizing antibodies to SARS‐CoV‐2 infection persists for months. Science, 370, 1227–1230. 33115920 10.1126/science.abd7728 PMC7810037

